# Development of a Spherical Model with a 3D Microchannel: An Application to Glaucoma Surgery

**DOI:** 10.3390/mi10050297

**Published:** 2019-04-30

**Authors:** Mahmoud Gallab, Seiji Omata, Kanako Harada, Mamoru Mitsuishi, Koichiro Sugimoto, Takashi Ueta, Kiyohito Totsuka, Fumiyuki Araki, Muneyuki Takao, Makoto Aihara, Fumihito Arai

**Affiliations:** 1 Department of Micro-Nano Mechanical Science and Engineering, Nagoya University, Furo-cho, Chikusa-ku, Nagoya, Aichi 464-8603, Japan; arai@mech.nagoya-u.ac.jp; 2 Japan Science and Technology Agency (JST), Chiyoda, Tokyo 102-8666, Japan; kanako@nml.t.u-tokyo.ac.jp; 3 Department of Mechanical Engineering, School of Engineering, The University of Tokyo, Bunkyo, Tokyo 113-8656, Japan; mamoru@nml.t.u-tokyo.ac.jp; 4 Department of Ophthalmology, School of Medicine, The University of Tokyo, Bunkyo, Tokyo 113-8656, Japan; ksugimoto007@gmail.com (K.S.); ueta816tky@gmail.com (T.U.); kiyototsuka916@gmail.com (K.T.); faraki-tky@umin.ac.jp (F.A.); mtakao-tky@umin.ac.jp (M.T.); aihara-tky@umin.net (M.A.)

**Keywords:** 3D microchannel, spherical model, blow molding, soft material, eye surgery simulator

## Abstract

Three-dimensional (3D) microfluidic channels, which simulate human tissues such as blood vessels, are useful in surgical simulator models for evaluating surgical devices and training novice surgeons. However, animal models and current artificial models do not sufficiently mimic the anatomical and mechanical properties of human tissues. Therefore, we established a novel fabrication method to fabricate an eye model for use as a surgical simulator. For the glaucoma surgery task, the eye model consists of a sclera with a clear cornea; a 3D microchannel with a width of 200–500 µm, representing the Schlemm’s canal (SC); and a thin membrane with a thickness of 40–132 µm, representing the trabecular meshwork (TM). The sclera model with a clear cornea and SC was fabricated by 3D molding. Blow molding was used to fabricate the TM to cover the inner surface of the sclera part. Soft materials with controllable mechanical behaviors were used to fabricate the sclera and TM parts to mimic the mechanical properties of human tissues. Additionally, to simulate the surgery with constraints similar to those in a real operation, the eye model was installed on a skull platform. Therefore, in this paper, we propose an integration method for fabricating an eye model that has a 3D microchannel representing the SC and a membrane representing the TM, to develop a glaucoma model for training novice surgeons.

## 1. Introduction

To practice surgical procedures before they are used on patients, it is necessary to have access to artificial [1] or animal models. Apart from the bioethical considerations, animal models have a number of disadvantages, such as different anatomical properties from those of humans, and their expensive and labor-intensive preparation. Subsequently, it is difficult to guarantee the reproducibility of the results of training or the evaluation of medical devices using animal models. Using artificial simulators is considered an ideal solution [2]. Artificial models are particularly useful for eye surgery because miniaturized surgical devices are often used.

Among eye pathologies, glaucoma is the second leading cause of blindness worldwide [3]. The disease has a prevalence of 3.54% in the adult population aged 40–80 years, and 64.3 million people are affected by it [3,4]. In the normal eye, there is a liquid, secreted from the ciliary body of the uvea, called aqueous humor. It has a regular outflow path to keep the intraocular pressure (IOP) within the normal range, which is 20 mmHg or less. The aqueous humor provides a path between the crystalline lens and iris and flows to the angle structure. Then, it flows through the trabecular meshwork (TM) of the angle structure, reaches the Schlemm’s canal (SC), and flows out to the veins [5]. In glaucoma, the drainage of aqueous humor into the SC is hindered, leading to an increase in IOP and subsequent damage to the optic nerve, resulting in a progressive loss of sight. Previous studies showed that the main source for the elevation in IOP is the resistance of the TM to the outflow of aqueous humor to the SC [6,7,8,9,10]. Lowering the IOP is the only uncontroversial therapeutic strategy for reducing the risk of glaucomatous progression [11]. The surgery is usually reserved for advanced stages of the disease, when drugs and laser treatments fail to sufficiently reduce the IOP to control it. To address the prevalence of glaucoma, an extensive training program that allows surgeons to earn a high degree of skill and an understanding of the principles of the operation is in high demand.

Several systems that simulate various kinds of eye surgery are already in use [12,13,14]. However, these simulation systems lack important anatomical details, such as the 3D microchannel, which represents the SC, over the spherical shape of the eye, which is necessary for the simulation of glaucoma surgery. The importance of creating SC structure, in the simulation systems, comes from that the target area of TM, which will be removed by surgeons, should be over the SC structure to make an access to the SC as a way for the aqueous humor to lower IOP. Surgeons determine the target area of TM by identify the location of SC. Moreover, these simulation systems are often fabricated from materials whose mechanical properties are different from those of the target tissue. Consequently, for development of the current surgical and education models, a fabrication method for the 3D microchannel over the spherical shape and materials with controllable properties are required. Additionally, fabrication of 3D microfluidic channels over complex shapes can contribute in making inroads in biological research. An important application of 3D microfluidic channel over complex shapes, that stimulate blood vessel, includes studies on blood flow, which requires 3D geometries with biocompatible materials. Moreover, 3D microfluidic channel allows us to realize accurate manipulation and analysis of fluids, cells, drug screening, and medical procedures [15,16].

A number of microfabrication techniques for 3D microchannels have been produced over the years for various types of applications. Photolithography is employed as a microfabrication technique for creating microchannels. Photolithography and femtosecond laser exposure methods have been used to fabricate 3D microchannels that work as simulators for capillary vessels [17]. However, these techniques have a number of limitations for applications related to biological systems. For example, such techniques are intrinsically expensive processes. Moreover, the complexity of these processes also limits the flexibility and size of fabrication. Micromachining, soft lithography, and laser ablation are other techniques for fabricating microfluidic channels, which are used for large-scale replication and production [18]. However, those techniques suffer from a limited availability of biological materials.

In this paper, we present a simple microfabrication technique that allows for the rapid construction of a 3D microchannel on a spherical shape. We apply this technique to fabricate a 3D microchannel that represents the SC structure on the inside surface of an eye model. Additionally, we used a blow-molding method to fabricate the thin membrane covering the microchannel, which represents the TM. We introduced this model as a surgical simulator for the inside-out (ab interno) approach, which is a kind of minimally invasive glaucoma surgery (MIGS). In the fabrication of the artificial eyeball and TM, we used materials with controllable properties to mimic those of human tissues. Finally, by mounting this glaucoma model on the eye surgery simulator (bionic eye surgery evaluator: Bionic-EyE^TM^) which was purpose-build by our group [19], a series of surgical exercises could be performed.

## 2. Materials and Methods

### 2.1. Design Concept

**Figure 1:**
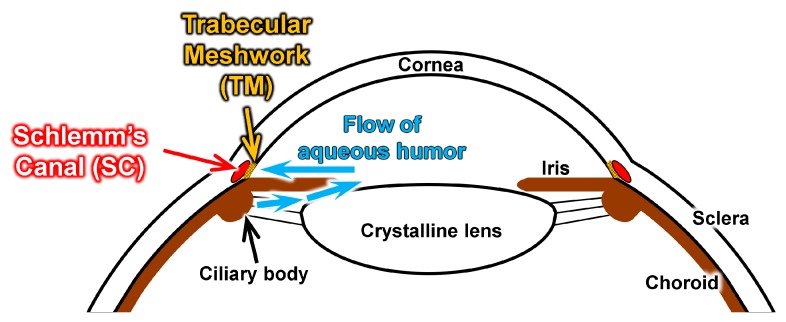
Schematic image of angle structure at corneal limbus and flow of aqueous humor in a human eye.

**Figure 2:**
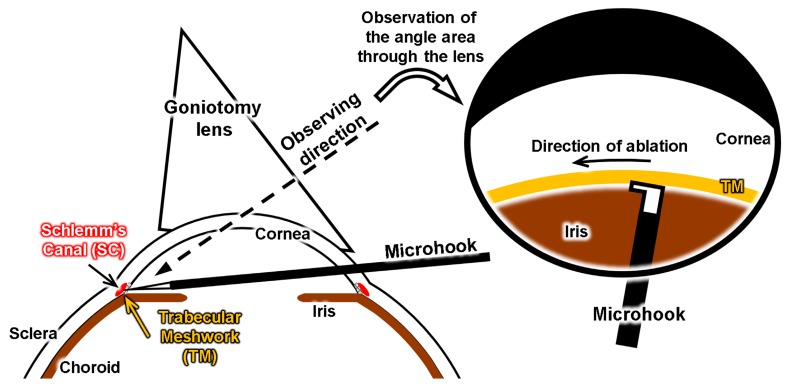
Concept of a glaucoma model for training on inside-out (ab interno) surgery.

An important structure for ab interno surgery is the angle structure around the limbus cornea, which is shown in Figure 1. The SC is a microchannel with a width of about 500 µm. The TM works as a filter during the flow of aqueous humor. A review of the surgical procedures of an ab interno approach using a microhook has been made [20]. Figure 2 shows a schematic of an inside-out approach for ablation of the TM. First, the surgeon tries to recognize the location of the SC using a goniotomy lens over the cornea. Then, they make an incision at the cornea limbus position to insert the microhook. Finally, they start to ablate part of the TM to allow the fluid inside the eye to exit through the SC, which lowers the IOP inside the eye. To perform such surgical exercises, we designed our eyeball model with an artificial sclera model, including a clear cornea part and 3D microchannel to represent the SC and TM, respectively.

Here, we discuss how to shape a 3D microchannel on the inner surface of a spherical shell that has a large curvature, representing an eyeball. By casting, we could easily mold the eyeball with a 3D microchannel, which works as an SC, on the curvature surface of the eyeball.

On the other hand, to fabricate an artificial TM with a thickness of 40 to 100 μm on a casted eyeball that has the microchannel, several fabrication methods can be considered, as shown in Table 1.

Therefore, in this study, an artificial TM, which is a lesion on glaucoma models, is achieved by the blow-molding method.

### 2.2. Fabrication of the Eyeball Model

**Figure 3:**
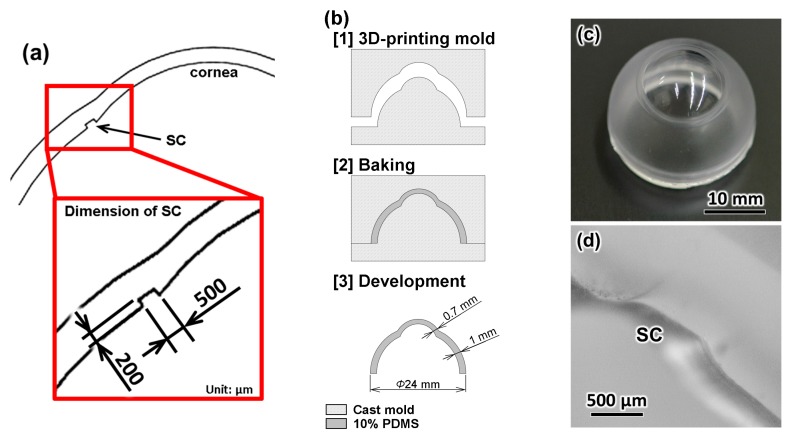
(**a**) Design and dimensions of eye model with 3D microchannel as artificial Schlemm’s canal (SC); (**b**) fabrication process of the eye model with SC; (**c**) fabricated eye model made from Polydimethylsiloxane (PDMS); (**d**) cross-sectional image of fabricated SC structure of the eye model.

Figure 3 shows the design and fabrication process for the eyeball model used in this study, which has a sclera model with a clear cornea and a 3D microchannel, which represents the SC. The thickness of the sclera and cornea, resulted from the casting process, designed to be 1 and 0.7 mm, respectively, to be similar to real tissues. Eye-shape cast molds were made in a precision-machined, three-dimensional mold. Polydimethylsiloxane (PDMS; Sylpot 184; Dow Corning Toray Co. Ltd., Tokyo, Japan) was mixed using a commercial manufacturing process, degassed, and poured into the mold. The polymer was cured by placing the mold in an oven at 120 °C for 2 h, after which it was removed from the hard mold.

### 2.3. Fabrication of the TM

**Figure 4:**
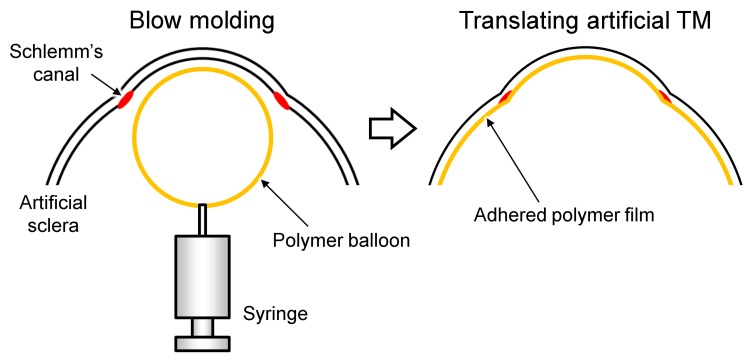
Fabrication process of an artificial trabecular meshwork (TM) adhered inside an artificial sclera.

The human TM is a thin membrane with a thickness over wide range of values (40–132 µm) [22]. Figure 4 shows the fabrication process of a TM using the blow-molding method. Polyvinyl chloride (PVC) (molecular weight 230,000, Sigma-Aldrich Co., St. Louis, USA) was the main polymer used in its fabrication. The PVC was dissolved, with a weight concentration of 30%, in a mixture of tetrahydrofuran (THF) (204-08745, Wako Pure Chemical Industries, Osaka, Japan) and N,N-dimethylformamide (DMF) (045-02916, Wako Pure Chemical Industries) (1:1), on a hotplate at 95 °C for 5 h. A syringe with a nozzle diameter of 10 mm was used to create a balloon from the PVC solution. The tip of the nozzle was immersed into the solution, and then the syringe was fed manually until the balloon was formed over the eyeball model.

Plasticizers are compounds that are added to brittle polymers such as PVC. They are expected to reduce the elastic modulus, tensile strength, and hardness of the polymer, while at the same time increasing its flexibility and elongation at breaking [23]. Di-(2-ethylhexyl) phthalate (DEHP) is the most commonly used phthalate plasticizer for plasticizing PVC. To control the mechanical behavior of PVC so that it mimics human tissue, DEHP (022-10815, Wako Pure Chemical Industries, Osaka, Japan) was mixed with the PVC solution at various concentrations. Then, the mechanical behavior of each concentration was tested.

### 2.4. Thickness Measurement of Artificial TM

To measure the thickness of the fabricated artificial TM over the eyeball model, we cut the eye model and used an optical microscope.

### 2.5. Measurement of the Mechanical Properties of TM

Uniaxial tensile testing was performed to failure with ten specimens tested for each concentration of the fabricated TM. The test specimens were prepared by spin-coating PVC/DEHP solution over a PDMS sheet at room temperature and waiting ten minutes for evaporation of the solvent. The test specimens were cut from the PVC/DEHP membrane (2 mm wide at their narrowest point with a gage length of 25 mm) using a JIS 7-type, dumbbell-shaped template (JIS K 6251) to assure uniformity and isolate the failure point away from the grips. The thickness of each sample was measured using a laser microscope. The uniaxial tensile test was performed on each sample using a tabletop EZ universal testing machine with a 50-N load cell (SHIMADZU, Kyoto, Japan) at an extension rate of 10 mm/min.

Polyvinyl chloride, a rubber-like material, has hyperelastic characteristics, showing a nonlinear relationship between the load and deformation. Many attempts have been made to theoretically reproduce the stress–strain curves obtained from experiments on the deformation of highly elastic rubber-like materials [24]. The elasticity of the artificial TM (E) was calculated as

$$\sigma =E\left(\lambda -\frac{1}{\lambda^{2}}\right)$$

where σ represents the uniaxial tensile stress and λ represents the strain ratio, which is defined as λ = 1 + ε with strain ε ≥ 0, respectively.

## 3. Results

### 3.1. Eyeball Model

Figure 3c shows the fabricated eyeball model made using PDMS. Polydimethylsiloxane is a popular polymer that has been used in building artificial organ models [17,25,26]. It has an elastic modulus of 1.32–2.97 MPa [27], very close to that of real sclera tissue (2 MPa) [28]. Moreover, PDMS shows excellent optical transparency. This facilitates a clear visualization of the SC through the cornea using a goniolens during the surgery simulation. To clarify the structure of the microchannel, which represents the SC, we made a cross section of the eye model. Figure 3d shows the resulting cross-sectional images.

### 3.2. Fabrication Results of the TM

**Figure 5:**
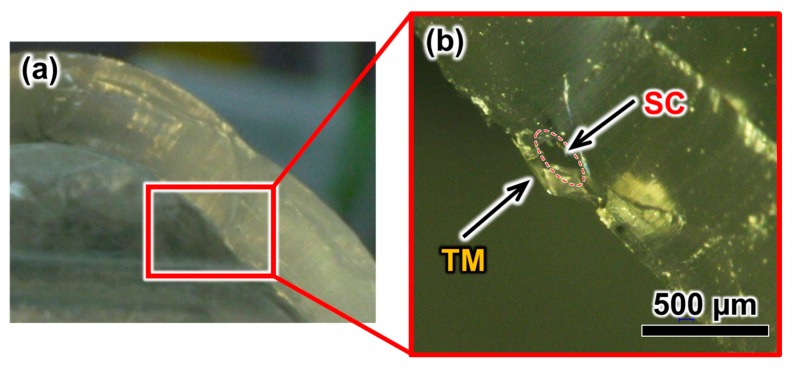
Sectional images of the fabricated eye model: (**a**) Cross-sectional image of the whole eye model; (**b**) high-magnification image of the angle for the tube structure of the SC covered with artificial TM.

To confirm the existence of the microchannel after forming the TM, we observed a cross section of the eyeball model. Figure 5 shows the cross-sectional images of the eyeball model after the artificial TM was formed. An advantage of blow molding compared with other methods, such as electrospinning and hydraulic transfer printing, is the reproducibility of the method. Additionally, the blowing method enabled us to form the TM while maintaining the structure of the microchannel. We could not prevent the microchannel from clogging when using the electrospinning or hydraulic transfer printing methods.

### 3.3. Evaluation of the TM Thickness

**Figure 6:**
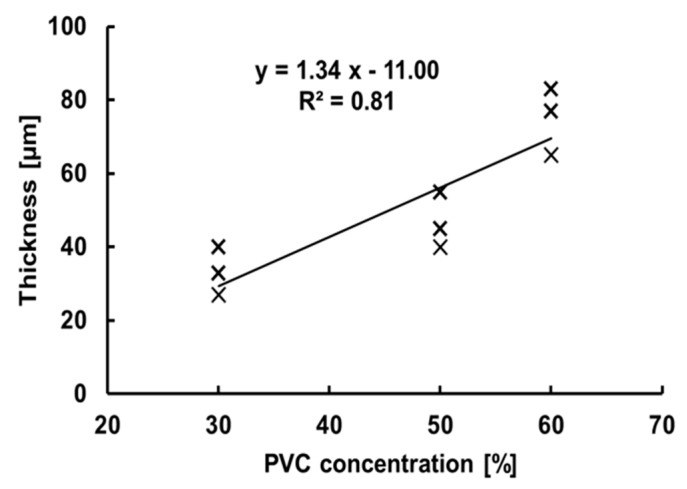
Relationship between the concentration of PVC solution and thickness of fabricated artificial trabecular meshwork.

Figure 6 shows the measured TM thickness as a function of the PVC concentration. We could control the thickness of the fabricated TM between approximately 20 and 80 µm by adjusting the concentration of PVC to solvent. The thickness of human TM has a wide range of values (40–132 µm) [22]. Therefore, PVC with a concentration of 30% to 60% should fall within the range of thickness of human TM.

### 3.4. Evaluation of Mechanical Properties of the TM

**Figure 7:**
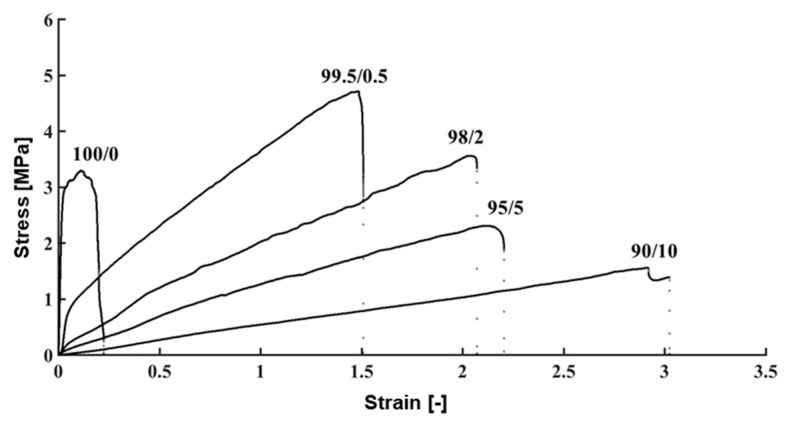
Stress–strain behavior of trabecular meshwork model. Numbers indicate the weight ratio of the PVC/DEHP mixture.

**Figure 8:**
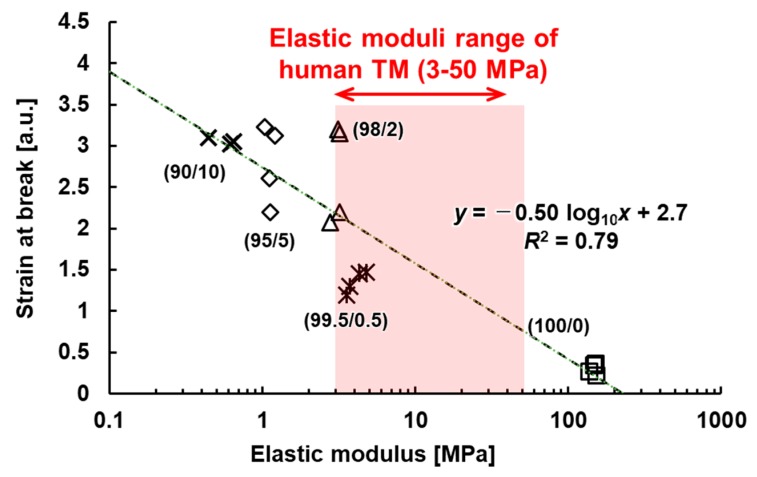
Elastic modulus and fracture strain of various concentrations of PVC/DEHP.

The elastic modulus of human TM, in both normal and glaucomatous eyes, was measured in previous studies, and the values were 0.228–1.085 MPa and 3–50 MPa, respectively [29,30]. Figure 7 shows the stress–strain behavior of the fabricated TM for various PVC/DEHP ratios. Table 2 summarizes the resulting elastic modulus, tensile strength, and strain at breaking for each mixture. We found that the elastic modulus of the PVC/DEHP (100/0) used in the fabrication of the TM was approximately three times the highest elastic modulus value of human TM tissue. By adding DEHP to the PVC solution, the elastic modulus of the fabricated TM could be tuned to match the elastic modulus of the human glaucoma TM. We could easily control the mechanical behavior of the fabricated TM using various concentrations of PVC/DEHP, as shown in Figure 8. The PVC/DEHP (99.5/0.5 to 98/2) ratio had an elastic modulus that fell within the wide range of elastic moduli of human TM tissue in an eye with glaucoma.

### 3.5. Simulation of Surgery with the Eye Model

**Figure 9:**
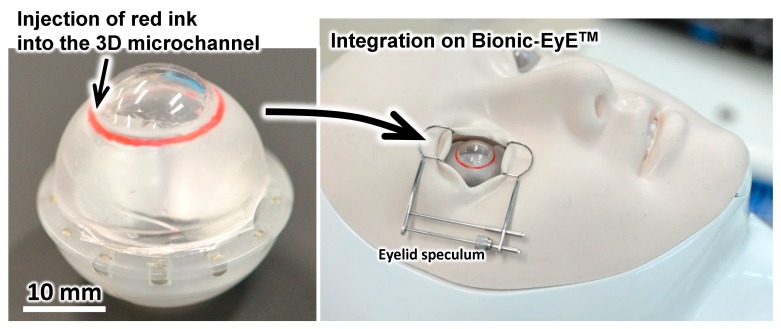
Photographs of the eye model installed on the Bionic-EyE^TM^ for simulation of micro invasive glaucoma surgery.

**Figure 10:**
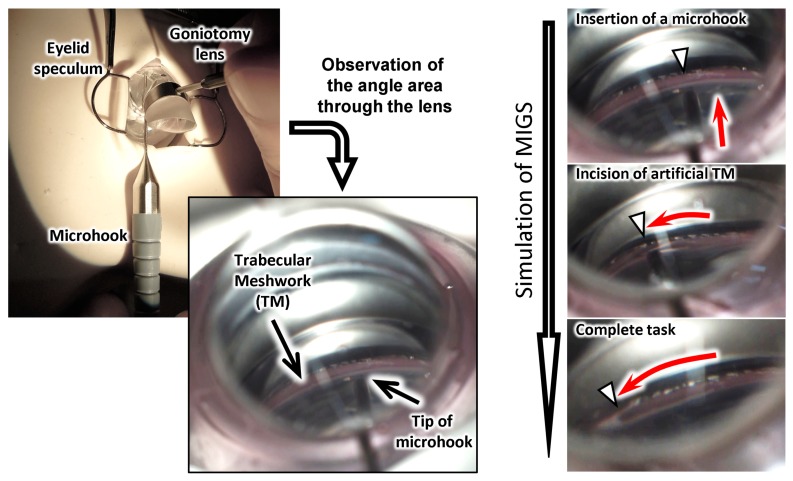
Photographs from the training of the micro-invasive glaucoma surgery with a microhook.

Figure 9 shows the artificial eye model mounted on the Bionic-EyE^TM^ to make the constraints of the surgery simulation similar to those in a real operation. Figure 10 and Supplementary Movie 1 show photographs and a movie of the training with our proposed model. First, the surgeon attempted, using a goniotomy lens over the cornea, to identify the location of the SC. Then, an incision was made at the cornea limbus position to insert the microhook. Finally, the surgeon could start to ablate part of the TM.

## 4. Discussion

We developed a simple technique to fabricate a 3D microchannel over a spherical shape, such as an eyeball. Additionally, we used a blow molding method to fabricate a thin membrane covering the 3D microchannel to represent the TM. We applied these techniques to fabricate an eye surgery simulator that comprised a sclera with a clear cornea and 3D microchannel to represent the SC and TM to simulate an ab interno approach, a kind of MIGS. The proposed eye surgery simulator accurately represents the eye’s shape and important anatomical structures, such as the SC and TM. The proposed fabrication methods, the 3D model made from a precision-machined, three-dimensional mold combined with polymer-molding, and the blow-molding process used for the eyeball and TM exhibited several advantages: (1) A complex 3D microchannel, representing the SC, can be accurately produced with our proposed method; and (2) it allows the use of a wide variety of materials (e.g., transparent materials and soft ones that mimic human tissues). In fact, using the proposed fabrication methods, we succeeded in fabricating an eyeball and TM with mechanical properties that approach those of human tissues. Moreover, the blow-molding method enabled us to fabricate a TM over the spherical shape of the eye with a thickness that could be controlled to that of real tissue.

The proposed eye model reported herein exhibited several advantages compared with animal models, such as reproducibility and longer durability. The simulator has been designed to duplicate, as closely as possible, the MIGS procedures encountered in clinical situations. These features are introduced through the proposed fabrication methods used in making the simulator. The proposed eye surgery simulator would facilitate the education of novice surgeons, allowing them to learn and practice basic skills for MIGS procedures while being evaluated objectively by an instructor.

One drawback of current fabrication methods is the syringe, used in the blow-molding of the TM, is fed manually. Therefore, even when we used the same concentration of PVC, the resulting thickness of the fabricated TM could have fairly large deviation, depending on the diameter of the balloon produced each time. However, from our TM measurements using the designed concentration ratio, the resulting thickness still fell within the range of human tissue, as shown in Figure 6.

The proposed model and the materials used in their fabrication may not perfectly represent all of the mechanical properties of human tissues (e.g., they exhibit higher tensile strengths and breaking strain values), as summarized in Table 2. However, they serve as examples to validate that the fabrication methods reported in this paper and various polymer materials and their mixtures can be used. Furthermore, the proposed eye model and the current materials used in their fabrication would allow novice surgeons to learn the basic skills of the surgery. A preliminary assessment of the proposed model was performed with experienced surgeons, as shown in Figure 9 and Figure 10. A preliminary assessment revealed that the model could help novice eye doctors learn the basic skills of the surgery. Therefore, we succeeded in developing a novel training model for glaucoma surgery.

In the future, to obtain more similar characteristics, we will conduct a face validity study and improve the material properties.

## 5. Conclusions

Simple techniques to fabricate a 3D microchannel and fabricate a thin membrane over a spherical shape were developed. Using these methods, 3D models were made using a precision-machined, three-dimensional mold. Additionally, using polymer molding and blow-molding methods, an eye model comprising a sclera with a clear cornea and 3D microchannel, which represent the SC, and TM was built. These methods are versatile because they replicate anatomical details with submillimeter resolution and permit a wide range of materials to be used. Additionally, we succeeded in installing the MIGS model into the Bionic-EyE^TM^ and operated similar to an actual surgery. The proposed model can be used as a simulator for training novice surgeons on MIGS. A preliminary assessment by eye doctors showed that the model can help novice eye doctors to develop the basic skills of the surgery. We foresee a number of applications for the proposed eye model, besides simulation and training for the surgery, including medical device testing and surgical planning.

## Figures and Tables

**Table 1:** Benchmark of fabrication methods to fabricate the artificial trabecular meshwork for a glaucoma model.

Method Property	Dip Coating ^1^	Electrospinning ^2^	Hydraulic Transfer Printing ^3^	Blow Molding ^4^
	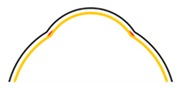	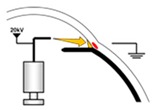	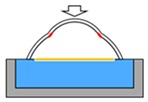	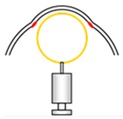
Uniformity of Thickness	-	✓	✓	✓
Reproducibility of Mechanical Properties	-	✓	✓	✓
Ease of Maintenance of SC Tube Structure	-	-	✓	✓
Adequateness of Thin Film Forming	-	-	-	✓

**^1^** Dip coating is a method of laminating a thin film onto the surface of a 3D object by immersing the object in a polymer solution. This method makes it somewhat easy to control the film thickness formed over the object. However, we cannot use it because the 3D microchannel on the eyeball, which represents the SC, will become clogged. **^2^** Electrospinning is a method of laminating a thin film with a micron- or submicron-scaled fiber using an electrical force to draw charged threads of polymer solution. This method is expected to allow ample adjustment of the film thickness and have good reproducibility. However, one of its major drawbacks is the difficulty in maintaining the groove structure of the SC. **^3^** Hydraulic transfer printing [21] is a method of transferring a thin film over 3D objects. This is done by floating the printed film on water and pressing the 3D object onto the film. Therefore, it is easy to adjust the thickness of the laminated layer, and the reproducibility of the mechanical properties is high. It is also expected that maintaining the groove structure of the SC would be easy. However, this method is difficult to laminate on a concave surface such as the inside of an eyeball model. **^4^** Blow molding is a widely used method in the molding of thin plastic containers. The main feature of this method is its high shape reproducibility even for concave shapes. This method is expected to be able to laminate the thin film covering the 3D microchannel without clogging it.

**Table 2:** Mechanical properties of Polydimethylsiloxane (PDMS) and Polyvinyl chloride (PVC)/Di-(2-ethylhexyl) phthalate (DEHP) used in this study to replicate sclera and trabecular meshwork tissues, respectively.

Tissues and Materials	Elastic Modulus (MPa)	Tensile Strength (MPa)	Strain at Breaking (mm/mm)
**Human Tissues**			
Sclera	2.0 [28]	-	0.2 [28]
TM Tissue (Normal Eye)	0.2–1.1 [29]	2.0 [29]	0.06 [29]
TM Tissue (Glaucomatous Eye)	3.0–52.6 [30]	-	-
**Artificial Materials**			
PDMS	1.3–2.9 [27]	3.5–7.6 [27]	-
PVC/DEHP (100/0)	147 ± 6	3.0 ± 0.3	0.30 ± 0.06
PVC/DEHP (99.5/0.5)	4.1 ± 0.5	4.0 ± 0.5	1.4 ± 0.2
PVC/DEHP (98/2)	3.1 ± 0.2	3.6 ± 0.9	2.7 ± 0.6
PVC/DEHP (95/5)	1.12 ± 0.06	2.3 ± 0.6	2.8 ± 0.5
PVC/DEHP (90/10)	0.6 ± 0.1	1.6 ± 0.2	3.1 ± 0.4

## Supplementary Materials

The following are available online at https://www.mdpi.com/2072-666X/10/5/297/s1, Video S1: Eye surgery simulator for an inside-out approach in the glaucoma surgery.
